# Hypoxia induces an endometrial cancer stem-like cell phenotype via HIF-dependent demethylation of SOX2 mRNA

**DOI:** 10.1038/s41389-020-00265-z

**Published:** 2020-09-11

**Authors:** Guofang Chen, Binya Liu, Shasha Yin, Shuangdi Li, Yu’e Guo, Mengfei Wang, Kai Wang, Xiaoping Wan

**Affiliations:** 1 Clinical and Translational Research Center, Shanghai First Maternity and Infant Hospital, Tongji University School of Medicine, Shanghai, China; 2 Department of Gynecology, Shanghai First Maternity and Infant Hospital, Tongji University School of Medicine, Shanghai, China

**Keywords:** Cancer stem cells, Cell signalling

## Abstract

Endometrial cancer stem cells (ECSCs) are stem-like cells endowed with self-renewal and differentiation abilities, and these cells are essential for cancer progression in endometrial cancer (EC). As hallmarks of the tumour microenvironment (TME), hypoxia and hypoxia-inducing factors (HIFs) give rise to the dysregulation of tumour stemness genes, such as SOX2. Against this backdrop, we investigated the regulatory mechanisms regulated by HIFs and SOX2 in ECSCs during EC development. Here, ECSCs isolated from EC cell lines and tissues were found to express stemness genes (CD133 and aldehyde dehydrogenase, ALDH1) following the induction of their ECSC expansion. Notably, m^6^A methylation of RNA and HIF-1α/2α-dependent AlkB homologue 5 (ALKBH5) participate in the regulation of HIFs and SOX2 in EC, as confirmed by the observations that mRNA levels of m^6^A demethylases and ALKBH5 significantly increase under hypoxic conditions in ECSCs. Moreover, hypoxia and high ALKBH5 levels restore the stem-like state of differentiated ECSCs and increase the ECSC-like phenotype, whereas the knockdown of HIFs or ALKBH5 significantly reduces their tumour initiation capacity. In addition, our findings validate the role of ALKBH5 in promoting SOX2 transcription via mRNA demethylation, thereby maintaining the stem-like state and tumorigenicity potential of ECSCs. In conclusion, these observations demonstrate a critical role for m^6^A methylation-mediated regulation of the HIF-ALKBH5-SOX2 axis during ECSC expansion in hypoxic TMEs.

## Introduction

Endometrial carcinoma (EC) is the most frequent gynaecological cancer in women, with an estimated 65,620 new cases and 12,590 deaths in the U.S. in 2020^1–3^. As the fourth most common cancer in women in terms of new cases, EC incidences have been rapidly increasing over the last 10 years as a consequence of a higher overall prevalence of obesity and metabolic syndromes^4,5^. However, the pathogenesis of EC remains poorly characterised.

Recent studies support the notion that a small subpopulation of EC cells, cancer stem-like cells (CSCs) or tumour-initiating cells contribute to self-renewal and differentiation in the early development of EC^6^. CSCs can be induced to maintain a stem-like state or to differentiate and express specific surface markers (i.e., CD24, CD34, CD38, CD44, CD117, CD55, CD133 and aldehyde dehydrogenase, ALDH1)^7–9^, among which CD44, CD55, CD117, CD133 and ALDH1 are reported to be enriched in ECSCs^6^. To date, the role of ECSCs is controversial, and no universal markers specific for ECSCs have been confirmed^10–12^.

Hypoxia is a vital niche characteristic for CSCs^13^, accelerating stem cell growth and tumour progression^14^. As master mediators of hypoxia, hypoxia-inducible factors (HIFs), including HIF-1α and HIF-2α, are indispensable for CSC activation and self-renewal and have been tightly linked to tumour malignancy^15^. HIFs mediate adaptive metabolic responses and mitochondrial ROS production in breast cancer tumour stem cells (BCSCs), enhancing the CSC phenotype via ITGA6 and promoting ALKBH5 expression, which plays a critical role in tumour-initiating breast cancer cells^16–18^. In addition, in mesenchymal stem cells, hypoxia induces interleukin-10 secretion, which in turn promotes CSC characteristics and lymphoma growth^19^. Similarly, HIFs participate in stem-like maintenance in various cancers, such as glioma stem-like cells^20^, prostate cancer stem-like cells^21^, hepatocellular cancer stem cells^22^, and liver cancer stem cells^23^. Thus, the identification and subsequent targeting of the molecular mechanisms driving hypoxia during CSC self-renewal provides a rational clinical strategy for cancer therapy.

CSCs can endure hypoxic conditions by rescheduling various stem genes involved in cell pluripotency, differentiation and proliferation^24^. Regarding core pluripotency factors, enhanced NANOG expression is observed under hypoxic stimulation within BCSCs^17^, while conditional SRY-box2 (SOX2) deletion in mice significantly represses the formation of skin squamous-cell carcinoma as a result of tumour regression^25^. Alternatively, in ME180 cells, discernible increased expression of SOX2 messenger RNA (mRNA) was identified under hypoxic conditions^26^, which is consistent with the evidence that in prostate cancer cells, enhanced SOX2 expression and HIF-1α- or HIF-2α-related phenotypes depend on the duration of exposure to hypoxia^27^. All these observations extend our knowledge regarding the link between hypoxia, HIFs and SOX2. Moreover, recent publications have demonstrated that changes in mRNA stability interfere with the expression of these pluripotency factors^17^. *N*^6^-methyladenosine (m^6^A) is an mRNA modification, and its addition to mRNAs is catalysed by methyltransferase-like 3 (METTL3), METTL14 and Wilms tumour 1-associated protein (WTAP)^28,29^. In gliomas, the methyltransferase METTL3 participates in SOX2 methylation modification and enhances SOX2 stability by binding the 3ʹUTR, thereby promoting the stem cell phenotype^30^. As an RNA demethylase involved in m^6^A modification, ALKBH5 is induced under hypoxic conditions^31,32^. To explore the molecular mechanisms that regulate m^6^A demethylation of SOX2 in ECSCs, we investigated the function of HIFs and SOX2 in ECSCs and demonstrated that ALKBH5 was induced under hypoxic conditions, wherein it decreased m^6^A methylation of SOX2 mRNA and changed the fate of ECSCs. This research establishes a possible continuum between the ECSC phenotype and m^6^A RNA methylation, which lays a foundation for the understanding of EC early development.

## Results

### The isolation and identification of ECSCs

**Fig. 1:**
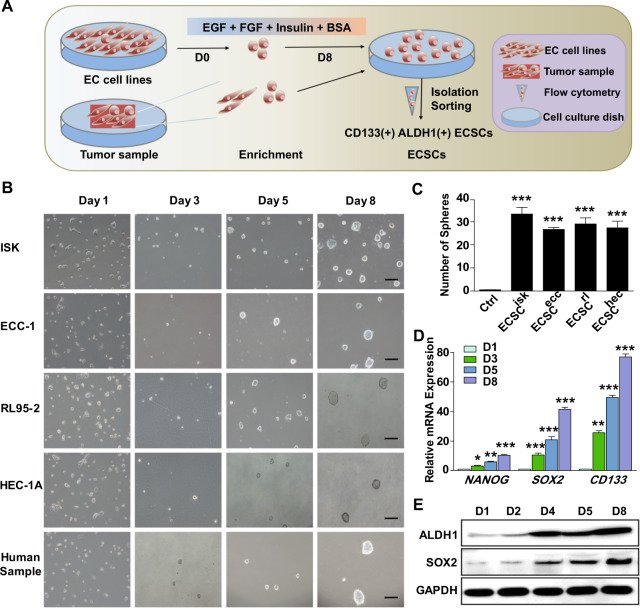
The isolation and identification of ECSCs by cell morphology and stemness gene analysis. **a** Schematic representation of the method of ECSC isolation. **b** Morphology of ECSCs isolated from different EC cell lines and human samples on days 1, 3, 5 and 8 (scale bar = 25 μm). **c** The numbers of spheres produced from different cells were analysed. **d** qRT-PCR analysis of stemness genes (*NANOG, SOX2* and *CD133*) in ECSC^isk^. Data are shown as the mean ± SEM (*N* = 3). *P < 0.05, **P < 0.01, and ***P < 0.001 vs. Day 1. **e** Immunoblotting findings of ALDH1 and SOX2 in ECSC^isk^ on days 1, 2, 4, 5 and 8.

To obtain ECSCs from EC cell lines and tissues, a new method was established in which ECSCs were isolated from EC cell lines and tumour samples; a schematic representation for the method is shown in Fig. 1a. In our study, ECSCs isolated from ISK, ECC-1, RL95-2, HEC-1A and human samples were described as ECSC^isk^, ECSC^ecc^, ECSC^rl^, ECSC^hec^ and ECSC^HM^, respectively. From day 1 to day 8, morphological changes in ECSCs were observed and recorded (Fig. 1b). On the first day, ECSC^isk^ were dispersed and flattened, and then a few stem cell colonies appeared on the third day; microscopy revealed that the cells gradually developed a uniform shape on the fifth day, and the cells could be continually subcultured (Fig. 1b). ECSCs from other EC cell lines and samples showed similar morphological changes over time. On the eighth day, colony accumulation increased, and sphere formation revealed a significant increase in all ECSCs. As analysed in Fig. 1c, compared to other ECSCs, ECSC^isk^ displayed the most spheres. To further confirm the characteristics of ECSC^isk^, the expression of stemness genes (*NANOG, SOX2* and *CD133)* and pluripotency markers (*ALDH1* and *SOX2*) was analysed by quantitative PCR (qPCR; (Fig. 1d)) and western blotting (Fig. 1e), respectively. As expected, the mRNA expression of *NANOG*, *SOX2* and *CD133* was significantly elevated over time, with the highest levels on day 8 in ECSC^isk^ (Fig. 1d), while other ECSCs showed comparable qPCR data (Supplementary Fig. S1A). In addition, immunoblotting findings of ALDH1 and SOX2 presented a trend of increasing with time in ECSC^isk^ (Fig. 1e). Since ECSC^isk^ showed the highest proliferation efficiency and the most stem cell-like state of the cells tested, they were selected for most of the subsequent experiments.

### Characterising the phenotype and pluripotency of ECSCs in vitro and in vivo

**Fig. 2:**
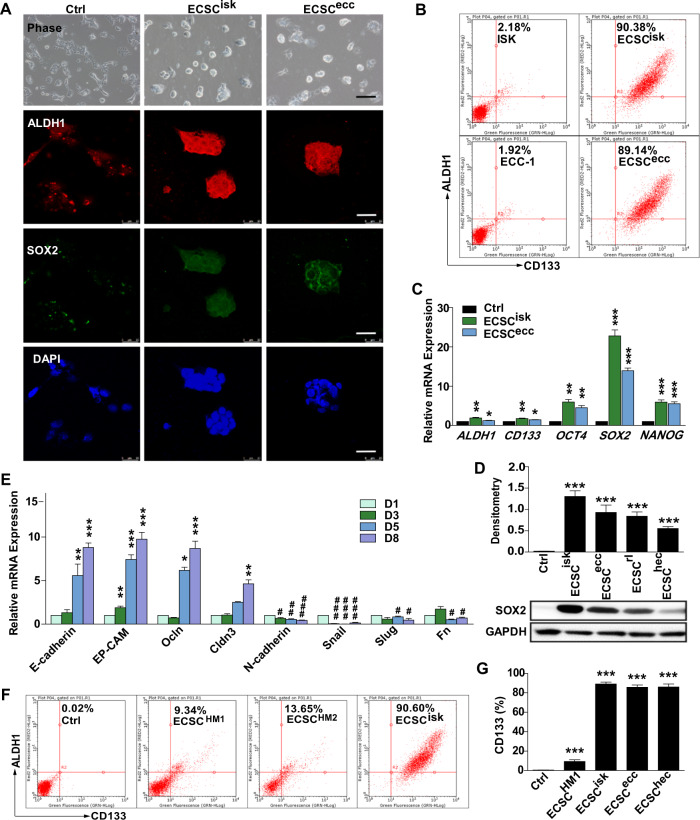
The qualification of ECSC pluripotency. **a** Morphology of ECSC^isk^ and ECSC^ecc^ and immunofluorescent staining of ALDH1 and SOX2. **b** FACS detection of ALDH1 and CD133 in ECSC^isk^ and ECSC^ecc^. **c** qRT-PCR analysis of stemness genes (*ALDH1, CD133, OCT4, SOX2* and *NANOG)* in ECSC^isk^ and ECSC^ecc^. **d** SOX2 protein expression by western blot in different ECSCs and control cells (ISK). **e** EMT gene expression in ECSC^isk^. **f** FACS data of the percentages of cells positive for ALDH1 and CD133 in two human samples and ECSC^isk^. **g** Statistics of the percentage of CD133-positive cells in ECSC^isk^. Mean ± SEM (*N* = 3). *^/#^*P* < 0.05, **^/##^*P* < 0.01, and ***^/###^*P* < 0.001.

To confirm the phenotype and pluripotency of ECSCs, confocal images of ALDH1 and SOX2 immunofluorescence staining revealed a uniform shape and phenotype of ECSC^isk^ and ECSC^ecc^ (Fig. 2a). Consistently, FACS analysis showed markedly higher percentages of ALDH1 and CD133 in ECSC^isk^ and ECSC^ecc^ than in ISK and ECC-1 cells (Fig. 2b). Alternatively, the mRNA levels of *ALDH1, CD133, OCT4, SOX2* and *NANOG* exhibited a discernible increase in ECSC^isk^ and ECSC^ecc^, especially SOX2 in ECSC^isk^ (Fig. 2c). Further immunoblotting data showed that SOX2 levels in different ECSCs were much higher than they were in control cells (Fig. 2d). Moreover, epithelial-mesenchymal transition (EMT) occurs as the epithelial cell markers *E-cadherin*, *EP-CAM*, *Ocln* and *Cldn3* decrease and the mesenchymal cell markers *N-cadherin*, *Snail*, *Slug* and *Fn* increase during long-term culture of ECSC^isk^ (Fig. 2e). FACS data revealed that the percentages of cells with stemness markers ALDH1 and CD133 were 9.34% and 13.65% in ECSC^HM1^ and ECSC^HM2^, respectively, while as a positive control, 90.60% of ECSC^isk^ were found to be positive for ALDH1 and CD133 (Fig. 2f). In addition, the percentage of CD133-positive cells was calculated to be higher in ECSCs than it was in ISK cells. Immunoblotting investigations into additional markers involved in ECSC^isk^ under differentiation conditions (ECSC medium removed of growth factors) showed that the levels of all stemness genes (SOX2, NANOG and ALDH1) decreased over time, while E-cadherin and Vimentin apparently increased after long-term culturing (Supplementary Fig. S2A). Similarly, the immunostaining of NANOG and SOX2 in ECSC^isk^ (Supplementary Fig. S2B) and ECSC^ecc^ (Supplementary Fig. S2C) was apparently reduced after differentiation for 4 days. Conclusively, these findings revealed that ECSCs might enhance stemness properties, spontaneously differentiating towards the epithelium in long-term culture and to develop the ECSC phenotype and pluripotency.

**Fig. 3:**
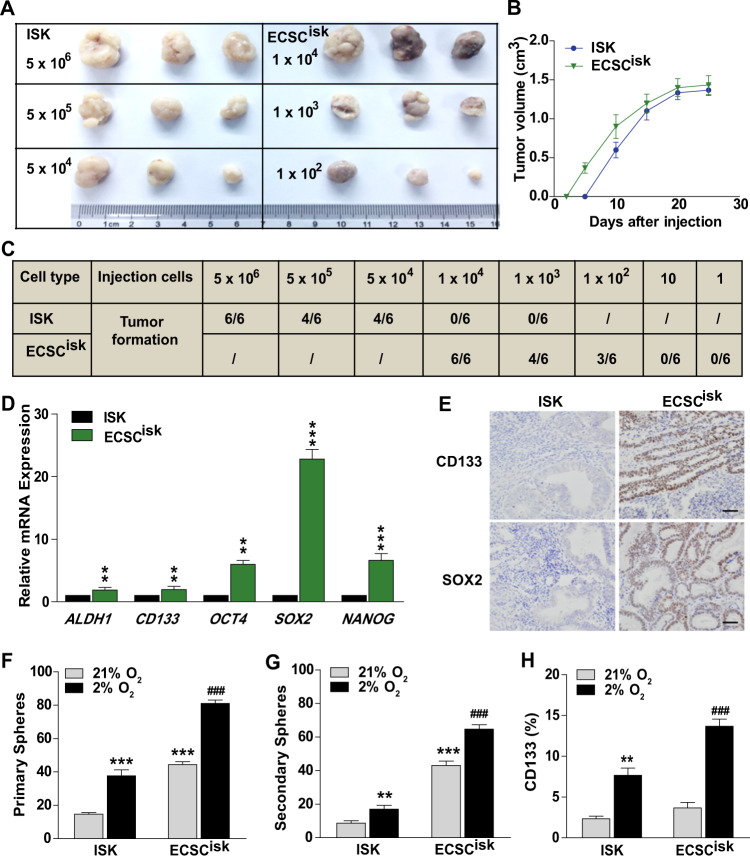
Characterisation of the increased stemness properties in ECSCs in vivo. **a**, **b** Tumorigenicity detected by the morphology (**a**) and volumes of xenografts (**b**) formed from ISK cells and ECSC^isk^. **c** Tumorigenicity initiating capacity of the minimum number of ISK cells and ECSC^isk^. **d** qRT-PCR analysis of the expression of *ALDH1, CD133, OCT4, SOX2* and *NANOG* in the tumours of the two groups. **e** Immunostaining of CD133 and SOX2 in tumours by IHC. **f**, **g** The numbers of primary (**f**) and secondary (**g**) spheres formed from ISK cells and ECSC^isk^ under hypoxic or normoxic conditions for 72 h. **h** FACS analysis of the percentages of CD133-positive cells in tumours from ISK cells and ECSC^isk^ in hypoxic or normoxic conditions for 72 h. **P < 0.01, and ***^/###^*P* < 0.001.

To test the tumorigenicity of ECSCs, we introduced ECSCs and ISK cells into xenograft mice model in a cell count-dependent manner (Fig. 3a). For forming a xenograft tumour, 5 × 10^4^ ISK cells for injection were needed, in contrast, only 1 × 10^2^ ECSC^isk^ cells were enough. The volumes of xenografts were visible on day 7 post ECSC^isk^ injection, while detectable tumours were observed on day 14 post ISK injection, markedly slower than the ECSC^isk^ group (Fig. 3b). The minimum required cell numbers of ISK and ECSC^isk^ for grafts formation were recorded in the Fig. 3c. Of note, pluripotency markers (*ALDH1, CD133, OCT4, SOX2* and *NANOG*) showed elevated mRNA levels xenografts in the ECSC^isk^ group (Fig. 3d), with stronger CD133 and SOX2 immunostaining (Fig. 3e). To further explore the stemness properties of xenografts in both groups, tumours were collected and digested into cell suspension, then the numbers of primary spheres were analysed after exposure to hypoxia or normoxia for 72 h (Fig. 3f). Interestingly, in both groups, the primary spheres of tumour cells under hypoxia were discernibly more than those under normoxia (Fig. 3f). With the primary spheres incubated in normoxia for 1 week, we compared the differences of secondary spheres between ISK and ECSC^isk^ xenografts, and similar results were obtained with the primary spheres (Fig. 3g). Consistently, under hypoxic conditions, both of ISK and ECSC^isk^ xenografts displayed higher percentages of CD133-positive cells, in which more CD133 expression was observed in ECSC^isk^ (Fig. 3h). Taken together, these results in vitro and in vivo support the notion that hypoxia plays a central role in the tumorigenicity of ECSCs.

### Hypoxia promotes the stem-like state of ECSCs

**Fig. 4:**
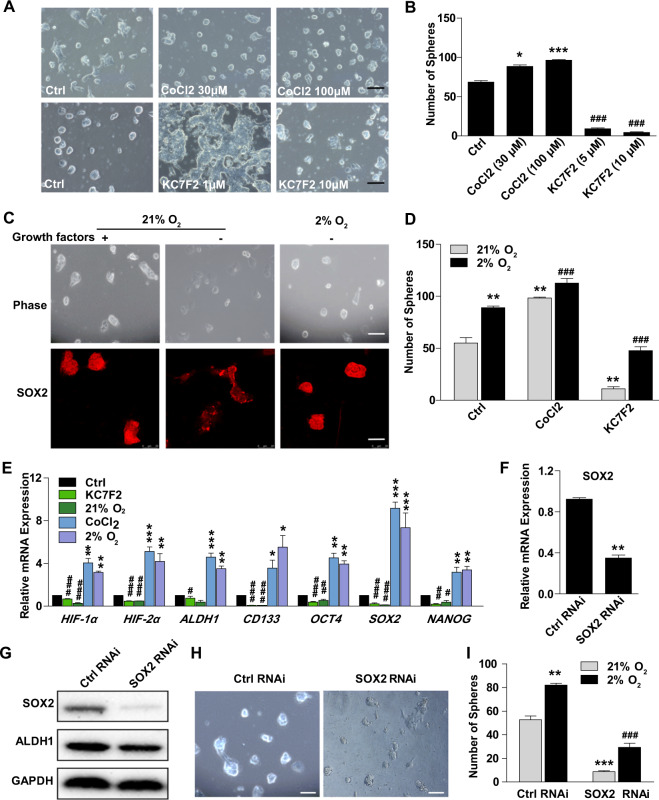
Hypoxia promotes a stem-like state in ECSCs. **a**, **b** Images showing the morphology (**a**) and sphere numbers (**b**) of ECSC^isk^ supplemented with CoCl_2_ (30 and 100 µM) or KC7F2 (1 and 10 µM). **c**, **d** Images showing the morphology of ECSC^isk^, immunofluorescent staining of SOX2 (**c**) and the number of ECSC^isk^ colonies (**d**) subjected to 2 or 21% O_2_ with or without growth factors. **e** mRNA expression analysis of *HIF-1α* and *2α* and stemness genes (*ALDH1, CD133, OCT4, SOX2* and *NANOG*) in ECSC^isk^ treated with CoCl_2_ (100 µM) or KC7F2 (10 µM) under normoxic or hypoxic conditions for 3 days. **f**, **g** qPCR (**f**) and immunoblotting (**g**) analysis of SOX2 after transfection with an siRNA. **h** Morphology pictures of ECSC^isk^ after 2 days of SOX2 inhibition in normoxic conditions (scale bar = 25 μm). **i** The ECSC^isk^ spheres with different SOX2 levels in hypoxic or normoxic conditions. Mean ± SEM. *^/#^*P* < 0.05, **^/##^*P* < 0.01, and ***^/###^*P* < 0.001.

The phenotype observed in ECSC^isk^ was promoted by CoCl_2_, which is a signal inducer of HIFs, in a dose-dependent manner; moreover, the addition of the inhibitor KC7F2 impeded the phenotype and increased the proportion of differentiated clones (Fig. 4a, b). Following analysis of the morphology, the numbers of ECSC^isk^ spheres were analysed upon activation of signal transduction via treatment with HIFs (Fig. 4b). In addition, the results of immunofluorescence staining in ECSC^isk^ revealed that when cultured in ECSC media deprived of growth factors, hypoxia induced a stem-like state with more SOX2 expression than was observed in normoxic conditions (Fig. 4c). As expected, the number of spheres from ECSC^isk^ was significantly increased in hypoxic conditions regardless of the presence or absence of HIF signal mediators (Fig. 4d). As shown in Fig. 4e, treatment with HIF signal inducers or hypoxia markedly reversed the expression of these pluripotency genes (ALDH1, CD133, OCT4, SOX2 and NANOG) in ECSC^isk^. It is clear that SOX2 is a core stemness transcription factor and mediates an early step in tumour initiation^25^. To determine the role of SOX2 in ECSCs, we explored variation in the stem-like phenotype in after changing SOX2 levels. After knockdown with RNA interference, SOX2 expression levels were confirmed by qPCR and western blot analysis (Fig. 4f, g) in ECSC^isk^. The images in Fig. 4h clearly show that the compacted colonial morphology disappeared by SOX2 inhibition when cultured in normoxia for 3 days. Additionally, more colonies were observed from ECSC^isk^ grown under hypoxic than normoxic conditions, while fewer colonies were observed after SOX2 inhibition (Fig. 4i). Consistent with previous data, the numbers of primary and secondary spheres were dramatically decreased by SOX2 inhibition, even under hypoxic conditions (Supplementary Fig. S3A, B), which was similar to the trend of CD133-positive cells (Supplementary Fig. S3C). Taken together, our data demonstrate that SOX2 is crucial for the expansion of hypoxia-induced ECSCs.

### HIFs are crucial for hypoxia-induced pluripotency in ECSCs

**Fig. 5:**
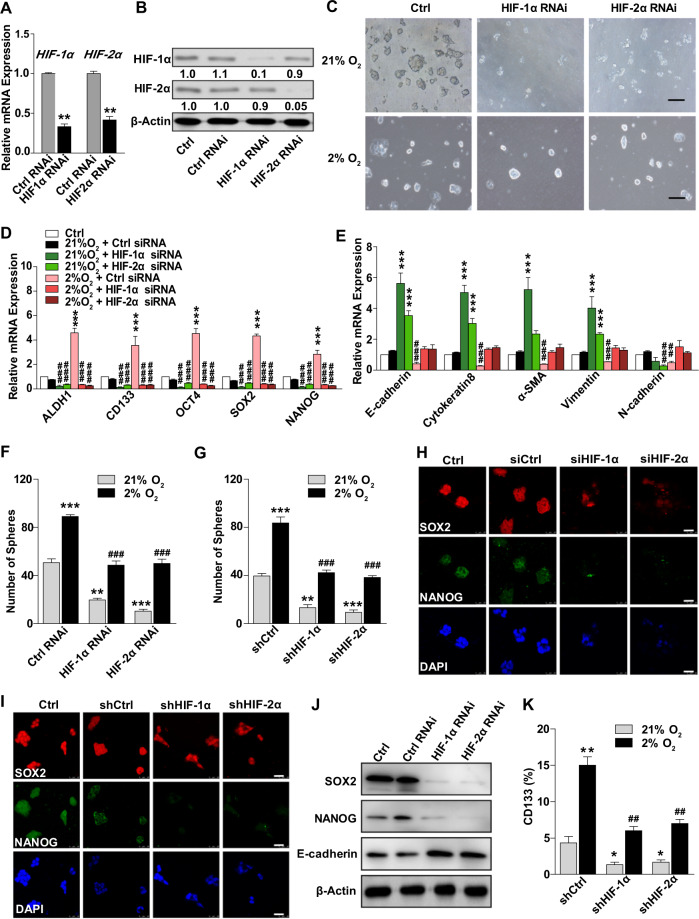
HIFs are required for hypoxia-induced SOX2 expression. **a**, **b** Validation of the siRNAs targeting HIF-1α and HIF-2α by qRT-PCR (**a**) and western blot (**b**). **c** Images of ECSC^isk^ morphology with low-HIF expression subjected to 2 or 21% O_2_. **d**, **e** qRT-PCR analysis of stemness genes (*ALDH1, CD133, OCT4, SOX2* and *NANOG*) (**d**) and lineage-specific genes (*E-cadherin, Cytokeratin8, α-SMA, Vimentin* and *N-cadherin*) (**e**) in ECSC^isk^ under different conditions, as shown in the panels. **f** The number of ECSC^isk^ colonies was calculated after transfection with a HIF siRNA. **g** The number of ECSC^isk^ colonies was calculated after transfection with a HIF shRNA. **h**, **i** Confocal images of SOX2 and NANOG in ECSC^isk^ subjected to various treatments. **j** SOX2, NANOG and E-cadherin protein levels were detected in ECSC^isk^ transfected with control and HIF siRNAs. **k** FACS analysis was used to determine the percentages of CD133-positive ECSC^isk^ under hypoxic or normoxic conditions. Scale bar = 25 μm. *P < 0.05, **^/##^*P* < 0.01, and ***^/###^ *P* < 0.001.

To further explore the molecular mechanisms of HIF signalling pathways involved in ECSC stemness, HIF-1α and HIF-2α were knocked down, and the efficiency was confirmed by siRNA and shRNA (Fig. 5a, b and Supplementary Fig. S4A, B). With HIF-1α or HIF-2α inhibition, ECSC^isk^ lost the compact morphology of their colonies; in contrast, the corresponding control ECSC^isk^ maintained stem-like morphology under normoxic conditions (Fig. 5c and Supplementary Fig. S4C). In addition, the reduced expression of pluripotency genes (*ALDH1*, *CD133*, *OCT4*, *SOX2* and *NANOG*) and the elevated expression of lineage-specific genes (*E-cadherin*, *Cytokeratin8*, *α-SMA*, V*imentin* and *N-cadherin*) were observed in both HIF-1α and HIF-2α knockdown ECSC^isk^ (Fig. 5d, e). Under hypoxic conditions, the number of spheres decreased after HIF-1α or HIF-2α inhibition, and there was less complete loss of positive colonies, while the sphere numbers increased in the control groups (Fig. 5f, g). Subsequent confocal images also revealed that HIF knockdown gave rise to low levels of NANOG and SOX2, decreasing the stem state of ECSCs (Fig. 5h, i). Moreover, after HIF-1α or HIF-2α inhibition in ECSC^isk^, Western blot analysis showed that SOX2 and NANOG decreased and E-cadherin increased (Fig. 5j), and the percentage of CD133-positive cells decreased regardless of whether the conditions were hypoxic or normoxic (Fig. 5k). These observations reveal that HIFs are required for hypoxia-induced pluripotency and a stem-like state of ECSCs.

### The effect of ALKBH5 on SOX2 expression and ECSC stemness

**Fig. 6:**
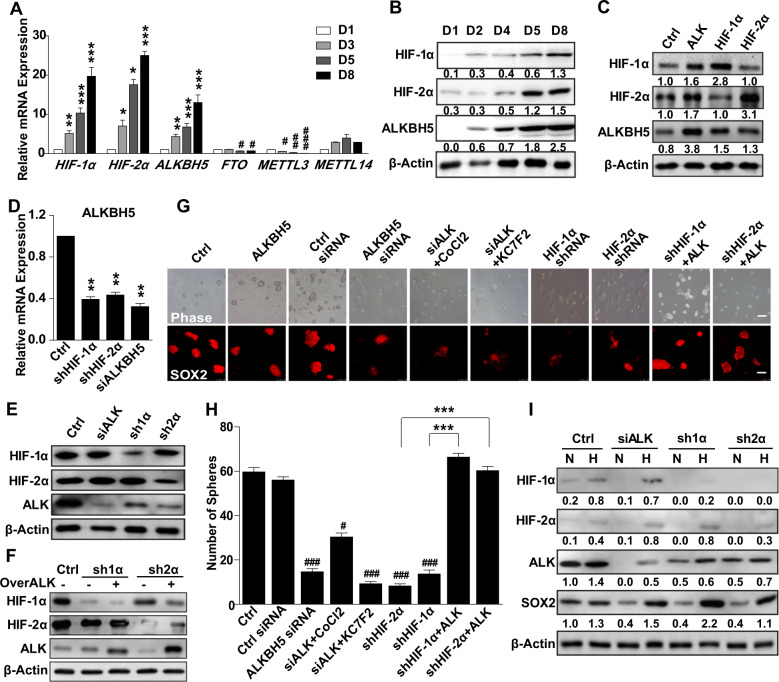
Knockdown of ALKBH5 decreases the stem-like state of ECSCs. **a**, **b** qPCR (**a**) and immunoblotting (**b**) analysis of ALKBH5 expression in ECSC^isk^ that were not passaged on days 1, 3, 5 and 8. The medium was changed every day. **c**, **d** After HIF overexpression or knockdown, the protein (**c**) and mRNA (**d**) levels of ALKBH5 were analysed. **e** ALKBH5 and HIF protein expression after ALKBH5, HIF-1α, or HIF-2α inhibition. **f** HIF-1α, HIF-2α, and ALKBH5 protein levels were detected in ECSC^isk^ in different co-transfection groups. **g** Images show the morphology, and confocal images reveal SOX2 in cells treated with the indicated siRNA or shRNA, supplemented with CoCl_2_ (100 µM) or KC7F2 (100 µM), or transfected with vector that overexpressed ALKBH5 (scale bar = 25 μm). **h** ECSCs under different treatments as shown were exposed to 21 or 2% O_2,_ and the number of spheres was determined. **i** Immunoblotting results for HIF-1α, HIF-2α, ALKBH5, and SOX2 in ECSC^isk^ with ALKBH5, HIF-1α, or HIF-2α inhibition. Mean ± SEM (*N* = 3). **P < 0.01, ***P < 0.001; ^#^*P* < 0.05, and ^###^*P* < 0.001.

As mRNA methylation and stability regulate the expression of pluripotency factors, we investigated the levels of both methylases (*METTL3/METTL14*) and demethylases (*FTO/ALKBH5*) in ECSCs; these factors sustain the mRNA methylation and stability of pluripotency factors. In ECSC^isk^ cells, *ALKBH5* clearly increased from day 1 to day 8 during the culture, while there were no discernible changes in *METTL14*, while *METTL3* and *FTO* were at low levels (Fig. 6a). qPCR analysis showed that *ALKBH5* was obviously upregulated (nearly 4-fold) after 5 days, implying that *ALKBH5* was the main demethylase involved in mRNA modification and stemness maintenance of pluripotency factors in ECSCs. In addition, during isolation and expansion of ECSC^isk^, both the mRNA and protein levels of ALKBH5 were markedly increased, which was consistent with HIF-1α and HIF-2α expression (Fig. 6b). The immunoblotting findings revealed that the ALKBH5 protein level was increased by HIF-1α or HIF-2α overexpression (Fig. 6c), while HIF knockdown decreased both the mRNA and protein levels of ALKBH5 (Fig. 6d, e). Additionally, knockdown of HIF-1α or HIF-2α significantly reduced the protein level of ALKBH5, which was reversed by ALKBH5 overexpression (Fig. 6f). ALKBH5 upregulation promoted SOX2 immunofluorescence staining regardless of HIF overexpression, which was consistent with the change in stemness morphology and sphere numbers of ECSCs, while ALKBH5 knockdown decreased SOX2 levels and stemness regardless of whether CoCl_2_ or KC7F2 were added (Fig. 6g, h). Moreover, as a potential mediator of ALKBH5, HIFs were found to be important for ECSC^isk^ in maintaining a stem-like state, which was observed by the fact that inhibition of HIFs significantly repressed SOX2 expression and the formation of ECSC spheres (Fig. 6g, h). Interestingly, compared to normoxic conditions, hypoxia promoted greater maintenance of SOX2 expression after ALKBH5, HIF-1α or HIF-2α inhibition (Fig. 6i), implying that hypoxia-induced ALKBH5 mediated the expression of SOX2. Based on these results, we presume that hypoxia induced ALKBH5 expression in a HIF-dependent manner in ECSCs.

### Hypoxia induces an ECSC stem-like state by demethylation of SOX2 mRNA

**Fig. 7:**
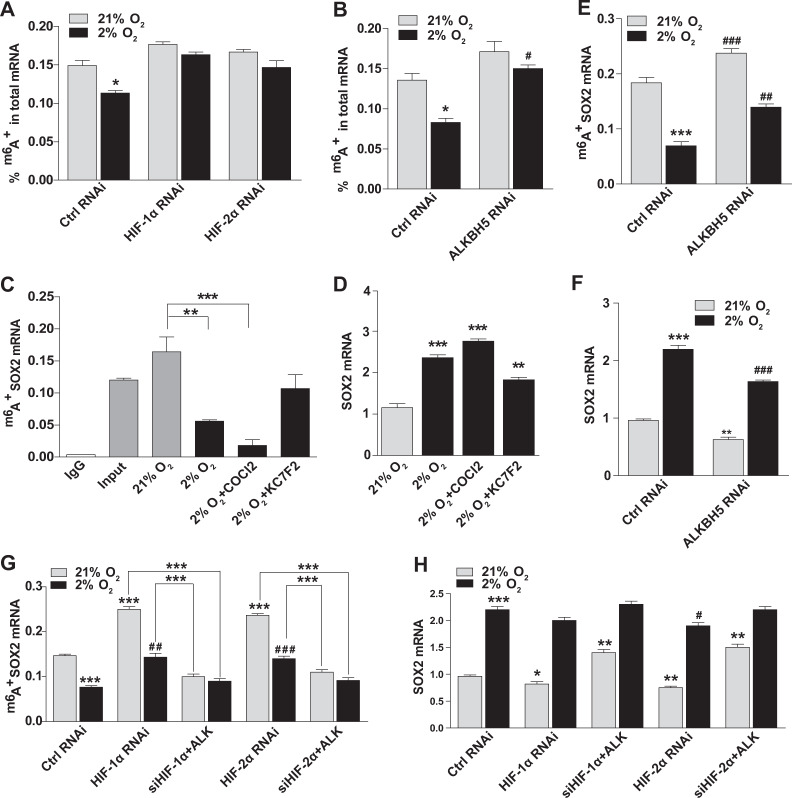
Hypoxia induces an ECSC phenotype via demethylation of SOX2 mRNA. **a**, **b** Total m^6^A levels in ECSC^isk^ after knockdown of HIFs or ALKBH5 were determined with an m^6^A RNA Methylation Quantification Kit. **c** RNA protein immunoprecipitation assays detected the level of m^6^A SOX2 mRNA in ECSC^isk^ under normal or hypoxic conditions for 48 h treated with CoCl_2_ (100 µM) or KC7F2 (100 µM), respectively. **d** *SOX2* mRNA levels in ECSC^isk^ treated with CoCl_2_ (100 µM) or KC7F2 (100 µM) were determined by qPCR. **e** RNA protein immunoprecipitation assay detected the level of m^6^A SOX2 mRNA in ALKBH5 knockdown ECSC^isk^ subjected to 21 or 2% O_2_ for 48 h. **f** SOX2 mRNA levels in shALK ECSC^isk^ were measured by qPCR. **g** Determination of the level of m^6^A SOX2 mRNA in HIF knockdown ECSC^isk^ with or without ALKBH5 overexpression that were also subjected to 21 or 2% O_2_ for 48 h. **h** qPCR analysis of *SOX2* mRNA in Ctrl and shHIF ECSC^isk^ under normoxic or hypoxic conditions for 48 h. Mean ± SEM (*N* = 3). **^/##^*P* < 0.01, and ***^/###^*P* < 0.001.

Here, we found that the hypoxia-induced demethylase ALKBH5 promoted SOX2 expression and the stem-like state of ECSCs. To further reveal the mechanisms of ALKBH5 and SOX2 in the modification of ECSCs, the m^6^A levels in total RNA were measured. With an m^6^A RNA methylation quantification kit, a significant decrease in m^6^A levels was observed under hypoxic conditions in control and ALKBH5 inhibited ECSC^isk^, while no obvious changes in HIF-1α or HIF-2α knockdown ECSC^isk^ were observed (Fig. 7a, b). Then, an RNA-binding protein immunoprecipitation (RIP) assay was performed to further assess whether ALKBH5 affected levels of m^6^A RNA modification. The data in Fig. 7c show that hypoxia led to decreased m^6^A modification of SOX2 mRNA in ECSC^isk^, regardless of whether they were treated with CoCl_2_; the decrease was not seen when ECSC^isk^ were treated with KC7F2 (Fig. 7c). In contrast, total SOX2 m^6^A levels significantly increased in ECSC^isk^ under hypoxic conditions with CoCl_2_ or KC7F2 treatment (Fig. 7d). Interestingly, the m^6^A levels of SOX2 mRNA were elevated by ALKBH5 inhibition under hypoxic or normoxic conditions (Fig. 7e), which was opposite of the trend observed for total SOX2 RNA (Fig. 7f). Subsequently, the m^6^A SOX2 mRNA and the total SOX2 mRNA in HIF-knockdown ECSC^isk^ were analysed, and the RIP results showed that ALKBH5 overexpression reversed the increase in m^6^A levels in SOX2 RNA caused by HIF knockdown under conditions with 2 or 21% O_2_ (Fig. 7g), while there was no significant change in total SOX2 RNA in HIF-knockdown ECSC^isk^ exposed to 2% O_2_ (Fig. 7h). These findings indicated that in hypoxic conditions, SOX2 mRNA was subject to HIF- and ALKBH5-dependent m^6^A demethylation.

**Fig. 8:**
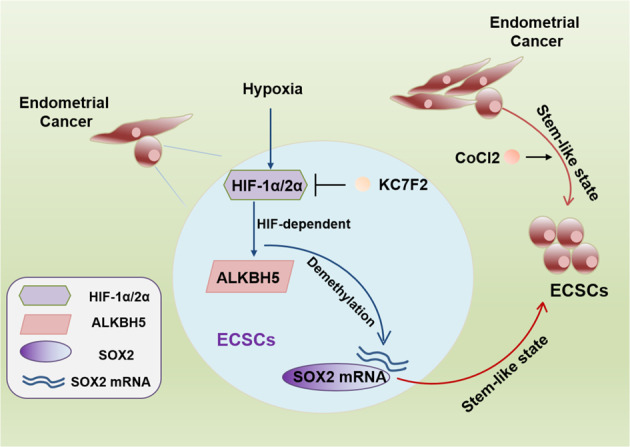
Graphical abstract of HIF-dependent demethylation of SOX2 mRNA in ECSCs. In ECSCs, HIF-1α and HIF-2α activate the expression of ALKBH5 under hypoxic conditions, facilitating SOX2 expression via demethylation of SOX2 mRNA. However, knockdown of HIF-1α and HIF-2α inhibits ALKBH5 and prevents the stem-like state of ECSCs, which is reversed by exposure to hypoxia or ALKBH5 overexpression. Blocking HIFs with KC7F2 inhibits the HIF/ALKBH5/SOX2 axis, which reduces the stemness of ECSCs, while activating HIFs with CoCl_2_ maintains an ECSC stem-like state. Collectively, hypoxia promotes the expression of the demethylase ALKBH5 and decreases m^6^A SOX2 by inducing demethylation of SOX2 mRNA, thus promoting the expression of SOX2 and the stemness of ECSCs.

In conclusion, with isolated ECSCs, we found that hypoxia promotes the sustained stem-like state and that HIF-1α and HIF-2α activate the expression of ALKBH5, thereby increasing SOX2 levels by decreasing m^6^A levels through the demethylation of SOX2 mRNA. Additionally, ALKBH5 knockdown inhibited SOX2 expression and ECSC stemness, supporting the idea that HIF-dependent ALKBH5 expression regulates m^6^A demethylation in total cellular RNA from ECSCs, thus promoting the expression of SOX2 and the ECSC phenotype (Fig. 8).

## Discussion

Our work resulted in establishing a method for isolating ECSCs from EC cell lines and primary human samples. It is quite remarkable that ECSCs undergo differentiation in vitro, and the maintenance of the ECSC phenotype relies on supplementation with many growth factors. Subsequent studies identified that hypoxia is the key extracellular stimulant in the TME that is required for the sustained phenotype and stem-like state of ECSCs. ECSC stemness can also be maintained via activation of the transcription factor SOX2. In addition, our findings demonstrate that m^6^A, the mRNA methylation modification mediated by methylases and demethylases, affects the maintenance of the ECSC state. This study uncovers a pivotal function of the demethylase ALKBH5 and provides valuable insights into the various roles of SOX2 m^6^A methylation in ECSCs. Thus, it is clear that HIF-dependent ALKBH5 expression influences ECSC expansion and stemness in hypoxic TMEs.

First, the ECSC isolation and cultivation method from previous studies that identified human mammary stems/progenitor cells was improved. The initial isolation methods have been developed to isolate different types of cancer stem cells from tissues, such as human breast cancer^33^, ovarian cancer^34^, choriocarcinoma^35^, head and neck squamous-cell carcinoma tumours^36^, colorectal cancer^37^, liver cancer^38^, and cervical cancer^39^. Some of the methods used for the isolation and identification of CSCs include primary extraction^40,41^, fluorescence activation^42^, magnetic activated cell sorting^43,44^, and side population selection^45^. Furthermore, serum-free suspension cultivation supplemented with growth factors is used to isolate CSCs^46,47^ from ovarian cancer^48^, glioma stem cells^49^, and cervical cancer^50^.

Second, our study identified that the ECSC phenotype and pluripotency are dramatically upregulated by hypoxia, which contributes to the characteristic stabilisation of HIFs in ECSCs. In various cancers, HIFs have been reported to enhance the CSC phenotype;^51–54^ on the other hand, hypoxia or HIFs enhance the dedifferentiation of CSCs^55^. Conversely, HIF-1α also promotes the differentiation of human or mouse embryonic stem cells (ESCs) into cardiac cells^56,57^. Moreover, hypoxia prevents vascular lineage differentiation through HIF1-mediated Oct4 suppression^58^. Our previous study demonstrated that hypoxia promotes the self-renewal of embryonic stem cells by activating HIF-1α and subsequently blocking VEGF secretion^59^. In addition, HIFs mediate ALKBH5 demethylation of NANOG mRNA and promote a BCSC phenotype^17^.

Third, our data demonstrated that the core stem gene SOX2 is the main target of HIF-dependent demethylase ALKBH5, which is involved in demethylation of downstream targets and further recruitment to m^6^A-modified sites. Our work in ECSCs revealed the relationship between HIFs and SOX2, which has been reported to be correlated with lymph node infiltration in EC^60^. In gastric cancer, SOX2 enhances HIF-1α promoter activity to regulate glucose metabolism^61^. Previous work on the crucial role of SOX2 in breast cancer cell migration showed that SOX2 upregulation in hypoxic conditions facilitated NEDD9 transcription and subsequent activation of HIF-1α expression^62^. This evidence extends our knowledge with regard to the diverse roles of SOX2 and HIFs in cancers.

In conclusion, our work improves the methods for isolation and cultivation of ECSCs from EC cell lines and human samples, characterising the stemness properties and facilitating xenograft tumour formation of ECSCs. We also uncovered a critical function of hypoxia and m^6^A methylation of SOX2 in EC. Activation of HIF signalling with CoCl_2_ or 2% O_2_ is capable of enhancing SOX2 transcription and maintaining ECSC behaviour in the ground state of pluripotency. Furthermore, HIF-dependent ALKBH5 expression increases in hypoxic TMEs, resulting in the demethylation of SOX2 mRNA and the maintenance of the ECSC phenotype; these data indicate that ECSCs maintain their stemness by activating the HIF/ALKBH5/SOX2 axis under hypoxic conditions. In the future, more delineation of the molecular mechanisms that regulate EC initiation and ECSC stemness is needed to better understand EC early development.

## Materials and methods

### Patients and tissue samples

EC specimens (*N* = 28) were collected from patients who underwent surgery or biopsy procedures at the Department of Gynecology of Shanghai First Maternity and Infant Hospital affiliated with Tongji University School of Medicine from April 2016 to December 2017. Patients who were pregnant, had known immunosuppressive diseases or were receiving immunosuppressive therapy, chemotherapy, radiotherapy, or other related antitumor therapies were excluded from the study. The patients’ information used are shown in Supplementary Table S1. The histology of all the specimens was confirmed by two independent pathologists. This study was approved by the Human Investigation Ethical Committee of Shanghai First Maternity and Infant Hospital, in accordance with the ethical standards as described in the 1964 Declaration of Helsinki and its later amendments or comparable ethical standards. Written informed consent was obtained from each participant after detailed explanations regarding the study objectives and procedures were provided.

### Cell culture

ISK, RL95-2, HEC-1A cell lines, and primary EC cells were cultured in DMEM/F12 medium (11960–044, Gibco, Carlsbad, CA, USA) supplemented with 10% FBS (HyClone) and 1% penicillin/streptomycin (15140–122, Gibco), while ECC-1 cells were cultured in RPMI-1640 medium (22400–089, Gibco) containing 10% FBS and 1% penicillin/streptomycin. Short tandem repeat (STR) analysis was performed in all cell lines. ECSCs were grown in DMEM/F12 media containing 20 ng/mL EGF, 10 ng/mL FGF, 20 ng/mL insulin, 0.4% BSA, and 1% penicillin/streptomycin. Under normoxic conditions, cells were cultured in an incubator with 5% CO_2_ and 95% air (21% O_2_). Under hypoxic conditions, cells were seeded in a modular incubator chamber (Billups-Rothenberg) and flushed for 2 min at 2 psi with a gas mixture containing 2% O_2_, 5% CO_2_ and 94% N_2_^63^. During culture, ECSCs were treated with CoCl_2_ (7646–79–9, Sigma-Aldrich, St. Louis, MO, USA) or KC7F2 (HY-18777, MedChemExpress, Monmouth Junction, NJ, USA) as noted in the experiments.

### Flow cytometry

ECSCs were isolated from the tumour sample, ISK, ECC-1, RL95-2, and HEC-1A cells. After 3 days of culture, primary spheres formed, and then the cell spheres were harvested and dissociated into single cells. Red blood cells were removed using red blood cell ACK lysis buffer (C3702, Beyotime). ECSCs blocked with 5% BSA in PBS before being suspended in a solution of 2% FBS in PBS, and then they were labelled with CD133 and ALDH1 antibodies. Intracellular staining was performed according to the instructions. The following antibodies were used: ALDH1A1 (#36671, 1:100, CST, Danvers, MA, USA), anti-rabbit IgG (H + L) (Alexa Fluor® 488 Conjugate) (#4412, 1:100, CST), CD133 (#60577, 1:50, CST), and anti-mouse IgG (H + L) (Alexa Fluor® 555 Conjugate) (#52286, 1:100, CST). Subsequent analysis was performed on a FACSCalibur cell analyser (BD Biosciences, San Jose, CA, USA) or a FACS Aria^TM^ II cell sorter (BD) according to the manufacturers’ standard operating procedures.

### Quantitative reverse transcription PC (qRT-PCR)

Total RNA was extracted using TRIzol^®^ RNA Isolation Reagents (Invitrogen, Carlsbad, CA, USA). Using SYBR green master mix (Takara, Dalian, Liaoning, PRC), mRNA expression analysis was detected on an ABI Prism 700 thermal cycler (Applied Biosystems, Foster City, CA, USA). All primers used are shown in Supplementary Table S2. The PCRs were performed in triplicate with at least three independent experiments.

### Western blot assay

Total cell lysates were collected in modified RIPA lysis buffer. Primary antibodies were used as follows: HIF-1α (ab6489, 1:1000, Abcam, Cambridge, MA, USA), HIF-2α (ab207607, 1:1000, Abcam), ALKBH5 (ab195377, 1:1000, Abcam), SOX2 (ab97959, 1:1000, Abcam), NANOG (ab109250, 1:1000, Abcam), ALDH1 (#36671, 1:1000, CST), E-cadherin (sc-21791, 1:1000, Santa Cruz), GAPDH (ab9485, 1:5000, Abcam) and ACTIN (AC026 1:5000, Abcam). HRP-conjugated anti-rabbit and anti-mouse antibodies were used, and chemiluminescent signals were detected with ECL Plus (Millipore).

### Transduction

All siRNA products targeting HIF-1α, HIF-2α and ALKBH5 were purchased from RiboBio (Guangzhou, PRC). For HIF knockdown, lentiviral vector FG12 (Addgene, Watertown, MA, USA) and packaging plasmids pRSV/REV, pMDLG/pRRE and pHCMVG were used. Recombinant lentiviruses that expressed shRNAs against coding regions of HIFs were produced in HEK-293T cells.

### Measurement of total m^6^A and m^6^A + SOX2 mRNA levels

Total RNA was extracted, and then m^6^A levels were determined. Using 200 μg aliquots of total RNA, an m^6^A RNA Methylation Quantification Kit (ab185912; Abcam) and an RNA-Binding Protein Immunoprecipitation Kit (17–700; Millipore, Burlington, MA, USA) were used according to the manufacturer’s instructions. To measure m^6^A + SOX2 mRNA levels, m^6^A immunoprecipitation was performed as described before^64^. A 1 μg aliquot of an m^6^A antibody was conjugated to a protein A-agarose slurry (Millipore) overnight at 4 °C. A 100 μg aliquot of total RNA was incubated with the antibody in immunoprecipitation buffer (50 mM Tris-HCl, 750 mM NaCl, and 0.5% Igepal CA-630) supplemented with RNase inhibitor for 3 h at 4 °C. RNA was eluted from the beads by incubation in 300 μL of elution buffer (5 mM Tris-HCl, 1 mM EDTA, and 0.05% SDS) with 4.2 μL of proteinase K for 1.5 h at 50 °C, and m^6^A + RNA was purified by phenol/chloroform extraction before being analysed by qPCR.

### Sphere assay

Cells were digested at 37 °C for 30 min to prepare single-cell suspensions, and then the cells were seeded in 6-well plates at a density of 5000 cells per well. After 3 days, the cells were photographed with an Olympus TH4-100 microscope, and primary and secondary spheres were counted.

### Mouse xenograft assays

Protocols were approved by the Animal Care and Ethics Review Committee and were in accordance with the Tongji University Guide for the Care and Use of Laboratory Animals. A method of randomisation was used to determine the experimental groups. In total, 78 female BALB/c nu/nu mice (4–6-week-old) were selected at random and were divided into different groups. A total of 5 × 10^6^ ISK cells or 1 × 10^4^ ECSC^isk^ were suspended in 100 μL of PBS and then were injected into the mice. After 2 weeks, the presence of tumours was examined. ISK cells (5 × 10^6^, 5 × 10^5^, 5 × 10^4^, 1 × 10^4^, or 1 × 10^3^) and ECSC^isk^ (1 × 10^4^, 1 × 10^3^, 1 × 10^2^, 10, or 1) were injected and analysed for their abilities to form xenograft tumours. After 4 weeks, subsequent experiments were performed.

### Statistics

Values are reported as the mean ± SEM. *P-*values were calculated by Student’s *t*-test. All graphs are plotted with GraphPad Prism 5 software.

## Supplementary information

Supplementary Information
