# Supplementary material for: Hypoxia induces an endometrial cancer stem-like cell phenotype via HIF-dependent demethylation of SOX2 mRNA
Source: Oncogenesis. 2020 Sep 11;9(9):81. doi: 10.1038/s41389-020-00265-z (PMC7484801; doi:10.1038/s41389-020-00265-z)
Supplement: Supplementary file 1 — Supplementary Information [file 41389_2020_265_MOESM1_ESM.docx]

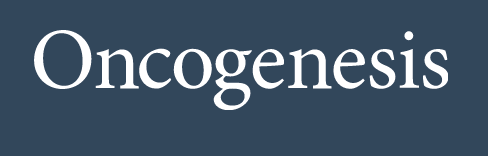


Supplementary Information for

**Hypoxia induces an endometrial cancer** **stem-like cell phenotype via HIF-dependent** **d****emethylation of SOX2 mRNA**

Guofang Chen^1, #, *^, Binya Liu^2, #^, Shasha Yin^1^, Shuangdi Li^2^, Yu’e Guo^1^, Mengfei Wang^1^, Kai Wang^1, *^, Xiaoping Wan^2, *^

# Guofang Chen and Binya Liu contributed equally to this work.

* To whom correspondence should be addressed.

E-mail: chenguofang@tongji.edu.cn (Guofang Chen), [kaiwangcn@yahoo.com](mailto:kaiwangcn@yahoo.com) (Kai Wang), wanxiaoping@tongji.edu.cn (Xiaoping Wan)

**This file includes:**

Figures S1 to S4 and Supplementary figure legends

Table S1 to S2 and Supplementary table legends

**S Figure legends**

**Figure S1. Stemness gene expression levels during ECSC expansion.**

(A) qRT-PCR analysis of stemness genes of ECSCs isolated from the EC cell lines ECC-1, RL95-2 and HEC-1A. Mean ± SEM; **P* < 0.05, ***P* < 0.01, and ****P* < 0.001.

**Figure S2. Characterization of the self-renewal and pluripotency of ECSCs.**

(A) Western blot analysis of pluripotency and lineage-specific markers of ECSC^isk^ after differentiation from days 1-20. (B, C) Immunofluorescence staining of pluripotency markers (NANOG and SOX2) in ECSC^isk^ (B) and ECSC^ecc^ (C). (D) A total of 1×10^6^ ISK cells or 1×10^4^ ECSC^isk^ were injected into female BALB/c nu/nu mice, and after 4 weeks, tumour formation in both groups was analysed (scale bar = 25 μm).

**Figure S3. The effect of SOX2 on the maintenance of ECSCs.**

(A, B) The number of primary (A) and secondary (B) mammospheres of ECSC^isk^ after SOX2 inhibition under hypoxic or normoxic conditions for 72 h was determined. (C) FACS analysis of the percentages of CD133-positive cells in tumours from SOX2 knockdown ECSCisk that were subjected to 2% or 21% O2 for 72 h. (D) Immunohistochemical staining of HIF1α and SOX2 in three different EC samples (EC sample #1, #2, #3) (scale bar = 25 μm). Mean ± SEM (*N* = 3). **^/##^ *P* < 0.01, and ***^/###^ *P* < 0.001.

**Figure S4.** **Knockdown of** **HIF-1α or HIF-2α represses the self-renewal of ECSCs.**

(A, B) qPCR (A) and Western blot (B) analysis of the expression of HIF-1α or HIF-2α following treatment with shRNAs. (C) The morphology of ECSC^isk^ after HIF-1α or HIF-2α inhibition under hypoxic or normoxic conditions for 2 days (scale bar = 25 μm). Mean ± SEM (N = 3). ***P* < 0.01.

**Figure S1**

**
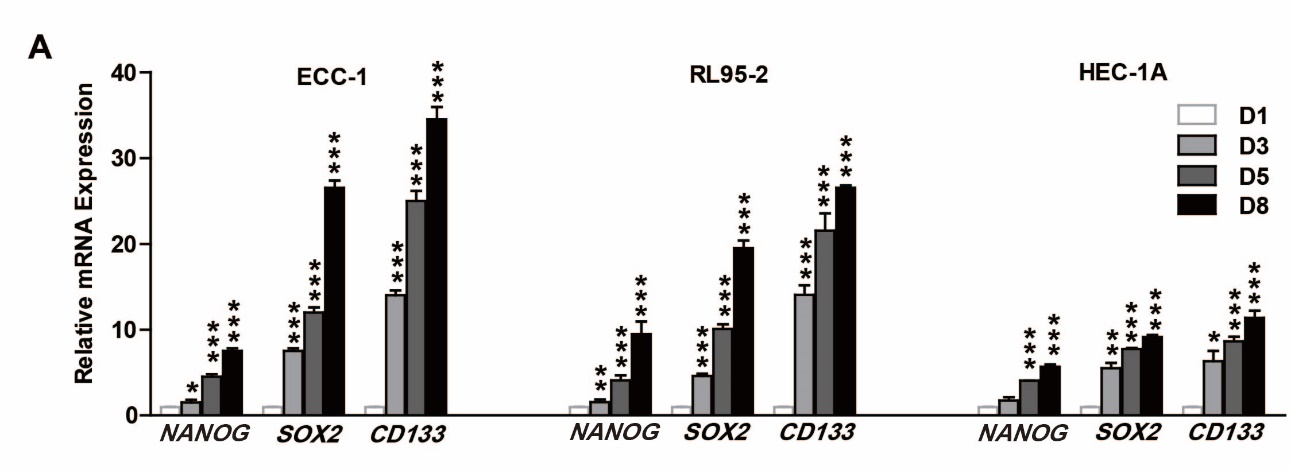
**

**Figure S2**

**
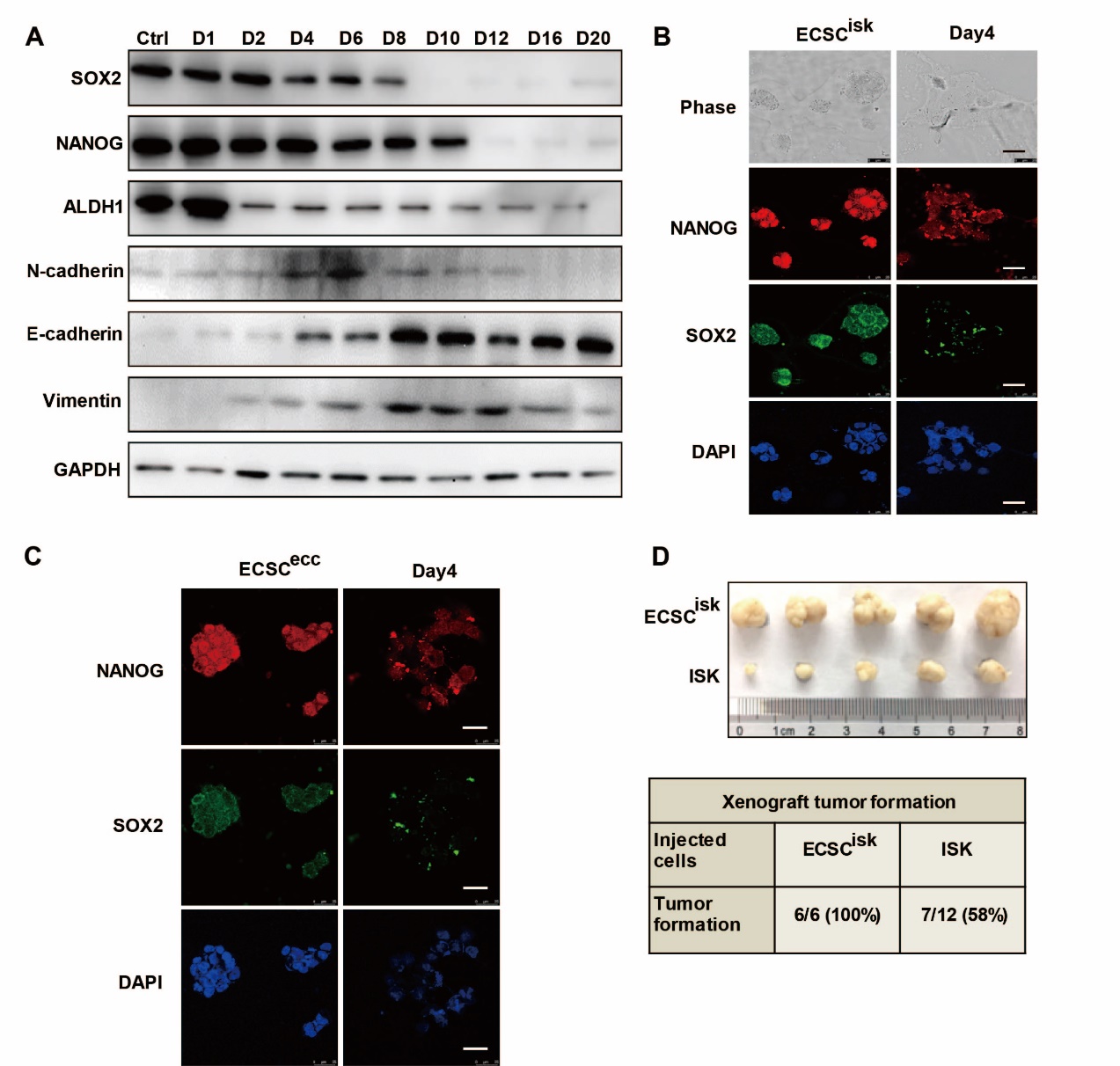
**

**Figure S3**

**
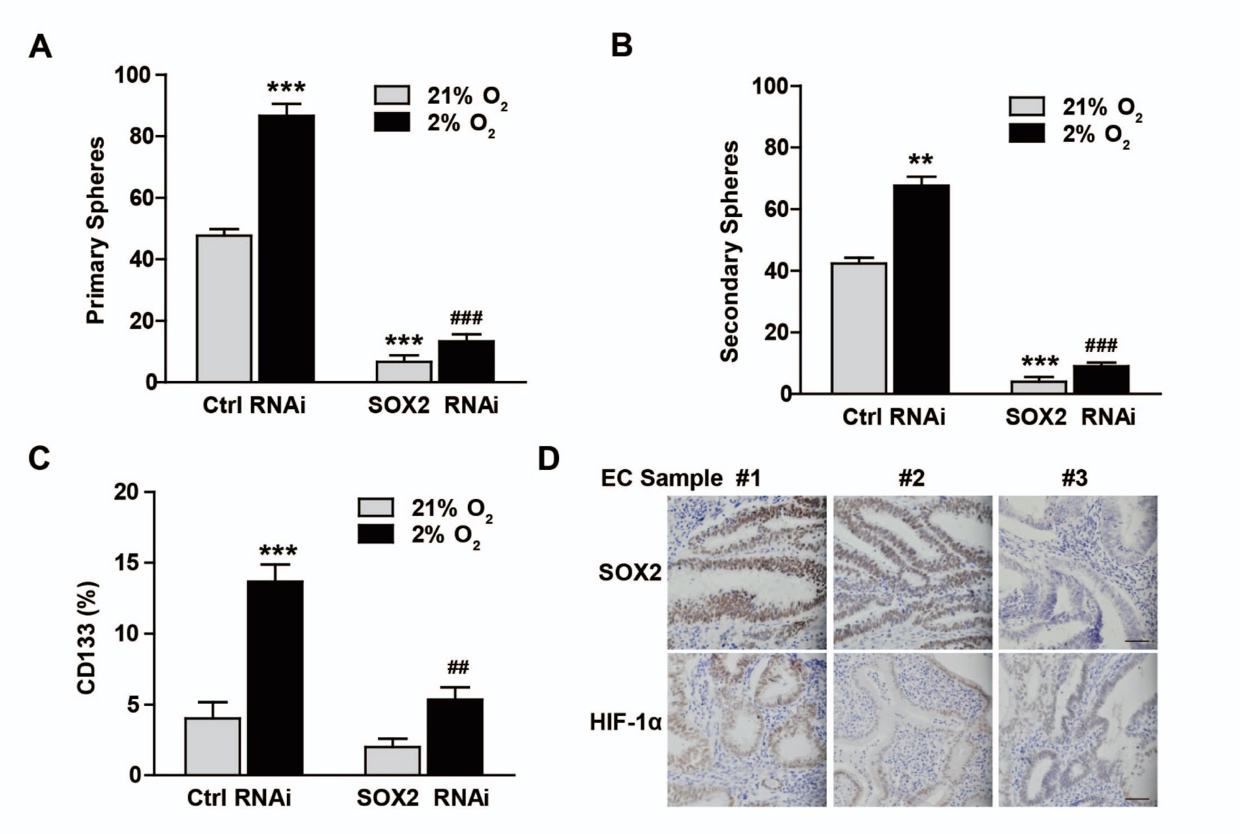
**

**Figure S4**

**
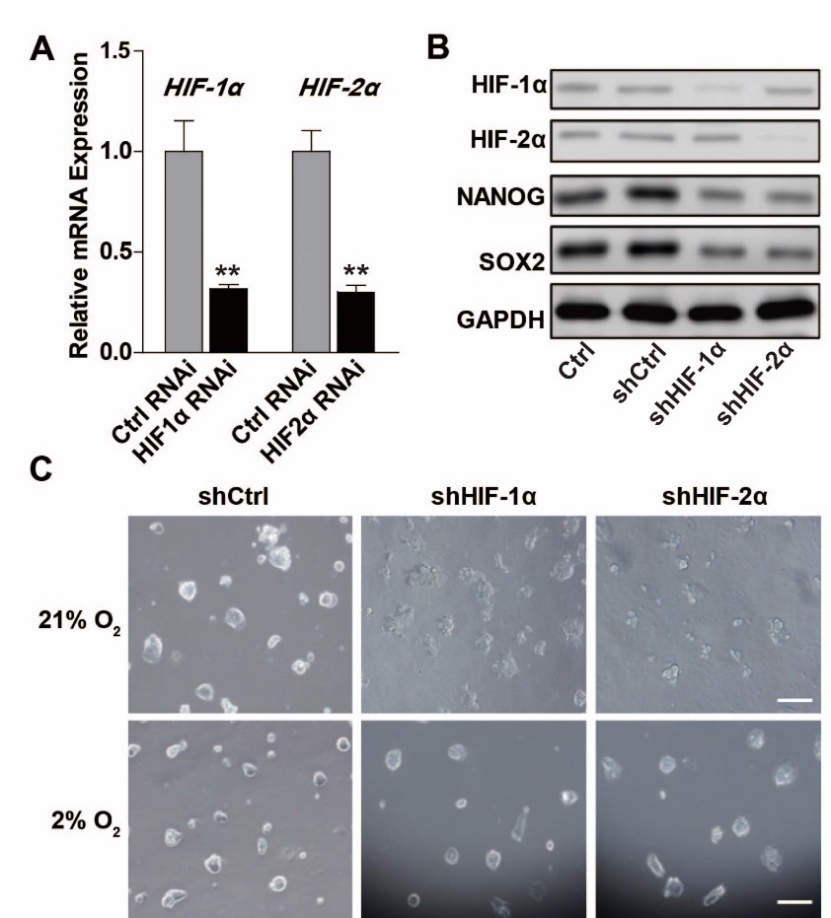
**

**S Table legends**

**Table S1. The correlation between the expression of SOX2 and HIF-1α with clinical features.**

Table S2. Primers sequences used for qRT-PCR analysis

**Table S1**

| **Characteristic** | **Total** | **SOX2 expression** | | **HIF-1α expression** | |
| --- | --- | --- | --- | --- | --- |
|  |  | **Low (*N* = 2)** | **High (*N* = 26**) | **Low (*N* = 7)** | **High** **(*N* = 21)** |
| **Age (years)** | 28 |  |  |  |  |
| ≤ 34 | 1 | 0 | 1 | 0 | 1 |
| 35-49 | 7 | 2 | 5 | 3 | 4 |
| ≥ 50 | 20 | 0 | 20 | 4 | 16 |
| **Histological differentiation** |  |  |  |  |  |
| Well/Moderate | 18 | 2 | 16 | 7 | 11 |
| Poor | 10 | 0 | 10 | 0 | 10 |
| **TNM stage** |  |  |  |  |  |
| I | 15 | 2 | 13 | 7 | 8 |
| II/III | 13 | 0 | 13 | 0 | 13 |
| **Based on mode** |  |  |  |  | |
| Curettage | 14 | 1 | 13 | 6 | 8 |
| Panhysterectomy | 14 | 1 | 13 | 1 | 13 |

Table S2

|  | Primer sequence |  | Primer sequence |
| --- | --- | --- | --- |
| HIF-1α | Forward 5’- ACCTATGACCTGCTTGGTGC -3’Reverse 5’- GGCTGTGTCGACTGAGGAAA -3’ | OCT4 | Forward 5’- GGTATTCAGCCAAACGACCA -3’  Reverse 5’- CACACTCGGACCACATCCTT -3’ |
| HIF-2α | Forward 5’- CATGCGCTAGACTCCGAGAACA -3’Reverse 5’- GCTTTGCGAGCATCCGGT -3’ | NANOG | Forward 5’- TTTGTGGGCCTGAAGAAAACT -3’  Reverse 5’- AGGGCTGTCCTGAATAAGCAG -3’ |
| ALKBH5 | Forward 5’- CGGCGAAGGCTACACTTACG -3’Reverse 5’- CCACCAGCTTTTGGATCACCA -3’ | MeTTL3 | Forward 5’- TTGTCTCCAACCTTCCGTAGT -3’  Reverse 5’- CCAGATCAGAGAGGTGGTGTAG -3’ |
| FTO | Forward 5’- GCTGCTTATTTCGGGACCTG -3’ Reverse 5’- AGCCTGGATTACCAATGAGGA -3’ | MeTTL14 | Forward 5’- TTTCTCTGGTGTGGTTCTGG -3’  Reverse 5’- AAGTCTTAGTCTTCCCAGGATTG -3’ |
| CD133 | Forward 5’- CAGAAGGCATATGAATCCAAAA -3’  Reverse 5’- ATAAACAGCAGCCCCAGGAC -3’ | E-cadherin | Forward5’- ATTTTTCCCTCGACACCCGAT -3’ Reverse5’- TCCCAGGCGTAGACCAAGA -3’ |
| SOX2 | Forward 5’- GACAGTTACGCGCACATGAA -3’ Reverse 5’- TAGGTCTGCGAGCTGGTCAT -3’ | EpCAM | Forward5’- TGATCCTGACTGCGATGAGAG -3’ Reverse5’- CTTGTCTGTTCTTCTGACCCC -3’ |

|  | Primer sequence |  | Primer sequence |
| --- | --- | --- | --- |
| Ocln | Forward 5’- ACAAGCGGTTTTATCCAGAGTC -3’Reverse 5’- GTCATCCACAGGCGAAGTTAAT -3’ | ALDH1 | Forward 5’- GCTCCATCATCTATCACCCGT -3’  Reverse 5’- ATCTCCGTGAATGAGGGTCCA -3’ |
| Cldn3 | Forward 5’- AACACCATTATCCGGGACTTCT -3’Reverse 5’- GCGGAGTAGACGACCTTGG -3’ | Cytokeratin8 | Forward 5’- CTGGTGGAGGACTTCAAGAAC -3’  Reverse 5’- GACCTCAGCAATGATGCTGTC -3’ |
| N-cadherin | Forward 5’- AGCCAACCTTAACTGAGGAGT -3’Reverse 5’- GGCAAGTTGATTGGAGGGATG -3’ | α-SMA | Forward 5’- GACGAAGCACAGAGCAAAAGAG -3’  Reverse 5’- TGGTGATGATGCCATGTTCTATCG -3’ |
| Snail | Forward 5’- ACTGCAACAAGGAATACCTCAG -3’ Reverse 5’- GCACTGGTACTTCTTGACATCTG -3’ | Vimentin | Forward 5’- TGGCACGTCTTGACCTTGAA -3’  Reverse 5’- GGTCATCGTGATGCTGAGAA -3’ |
| FN | Forward 5’- AGGAAGCCGAGGTTTTAACTG -3’  Reverse 5’- AGGACGCTCATAAGTGTCACC -3’ | m^6^A+ SOX2 | Forward 5’- TAATACGACTCACTATAGGGAGAAGTGAGGGAGCTCATAGGCCG -3’Reverse 5’-CAGAATCAAAATTCAGCAAGAAGCCTCTCCT -3’ |
| Slug | Forward 5’- TGTGACAAGGAATATGTGAGCC -3’ Reverse 5’- TGAGCCCTCAGATTTGACCTG -3’ |  |  |
